# Supplementary figures and images for: Estimating weekly excess mortality at sub-national level in Italy during the COVID-19 pandemic
Source: PLoS One. 2020 Oct 9;15(10):e0240286. doi: 10.1371/journal.pone.0240286 (PMC7546500; doi:10.1371/journal.pone.0240286)

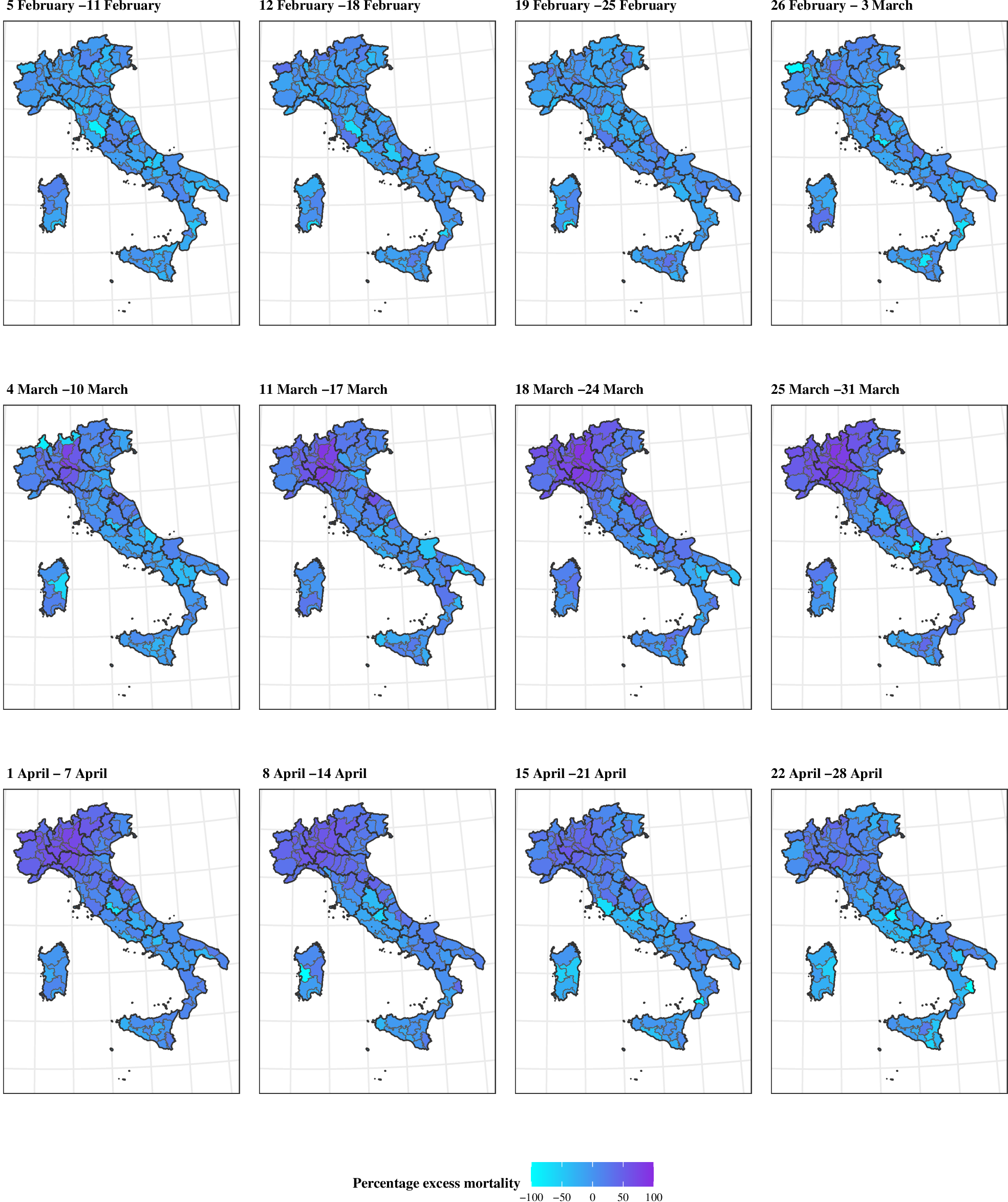

Supplement: S1 Fig — Weekly posterior predictive mean in 2020 for males. Period: 5 February–28 April. (TIF) [file pone.0240286.s001.tif]

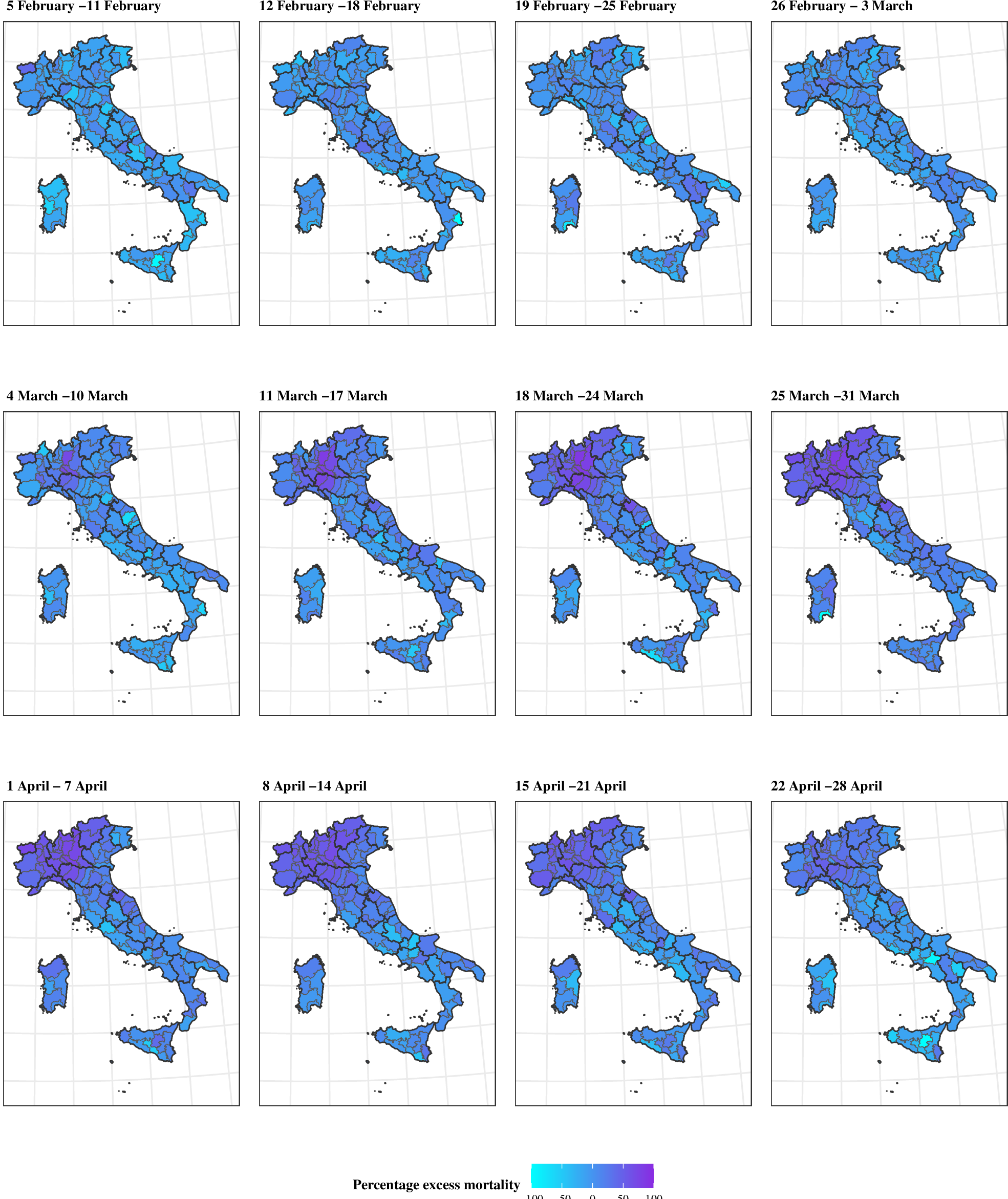

Supplement: S2 Fig — Weekly posterior predictive mean in 2020 for females. Period: 5 February–28 April. (TIF) [file pone.0240286.s002.tif]

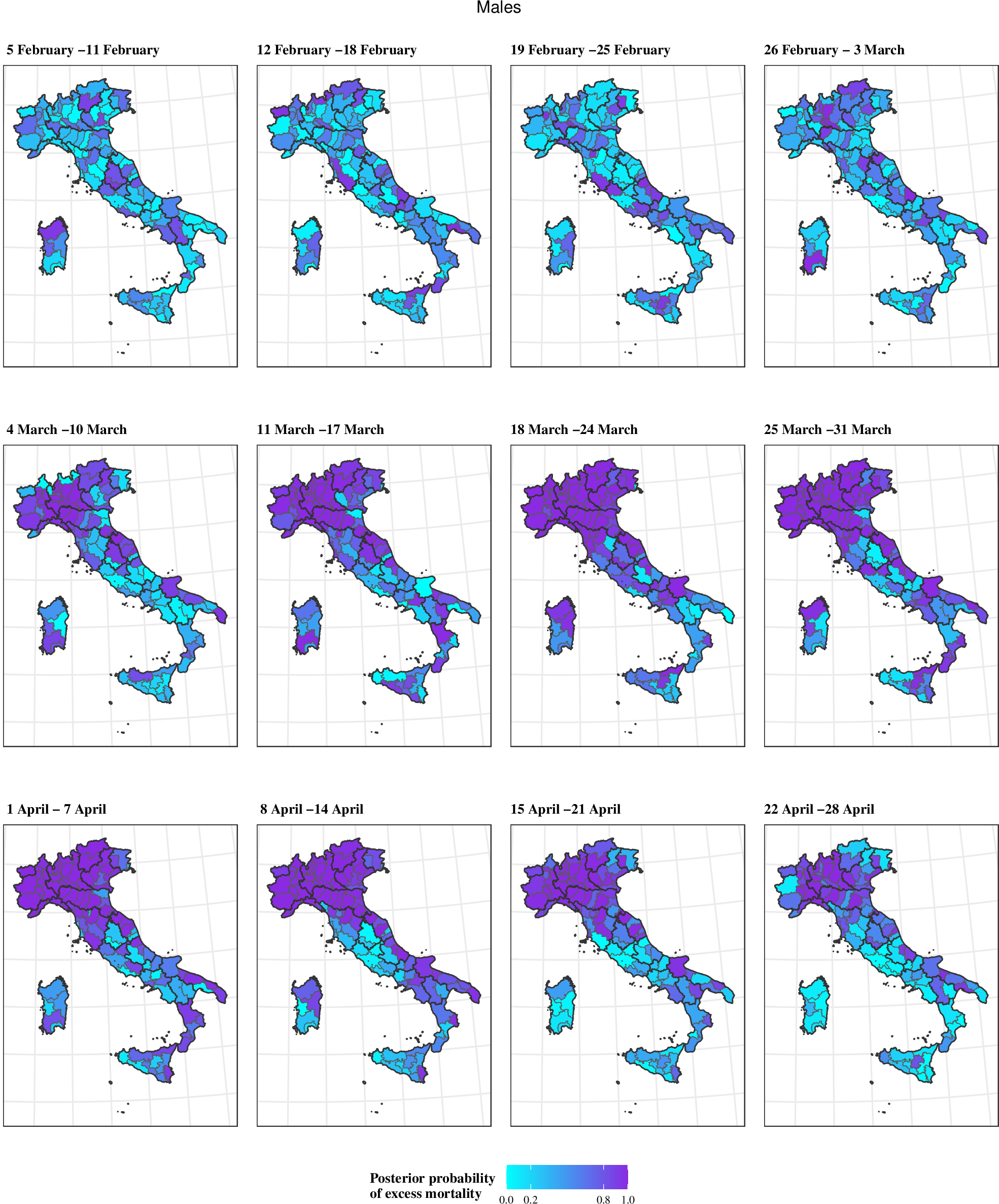

Supplement: S3 Fig — Weekly posterior mean in 2020 for males. Period: 5 February–28 April. (TIF) [file pone.0240286.s003.tif]

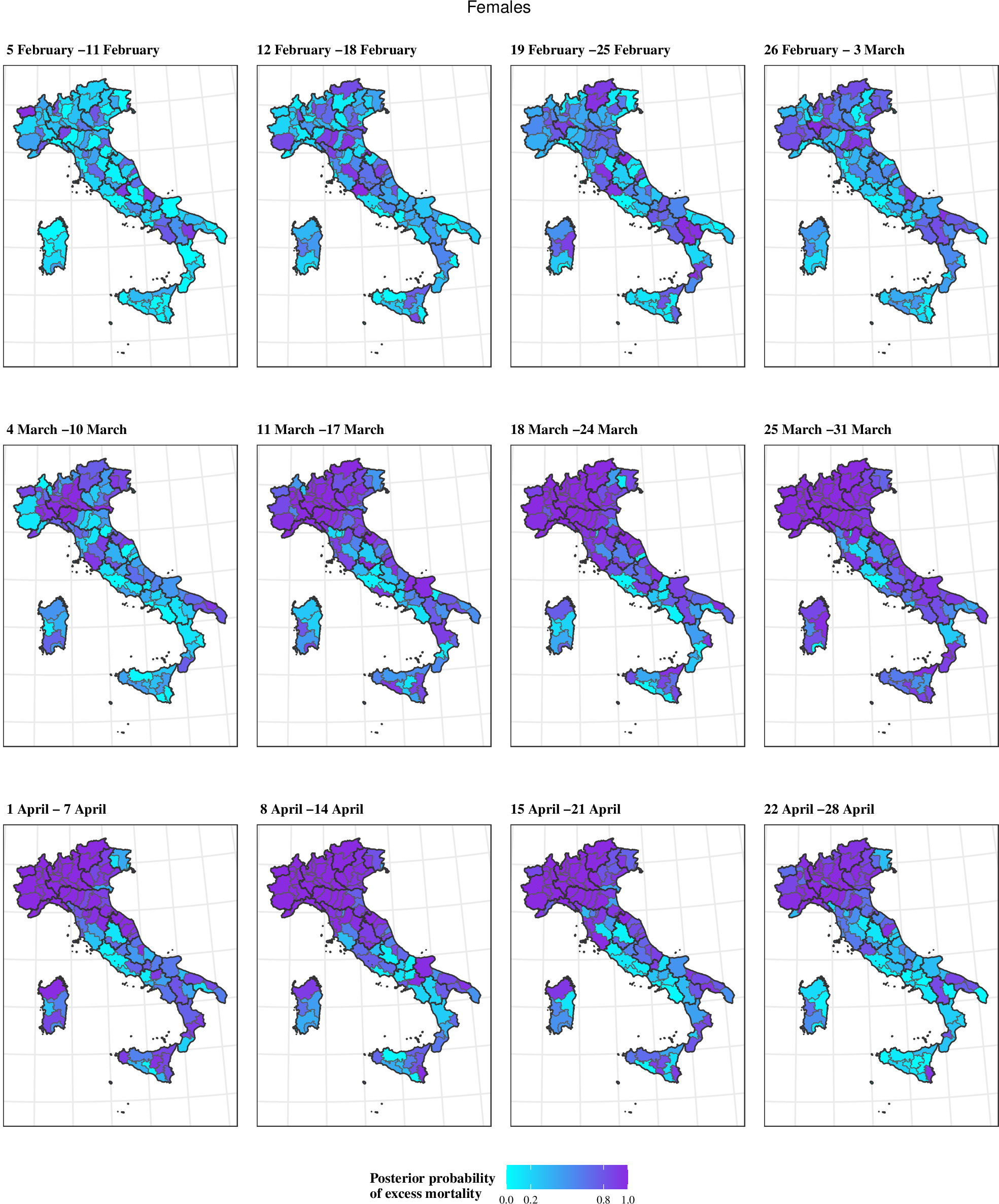

Supplement: S4 Fig — Weekly posterior mean in 2020 for females. Period: 5 February–28 April. (TIF) [file pone.0240286.s004.tif]

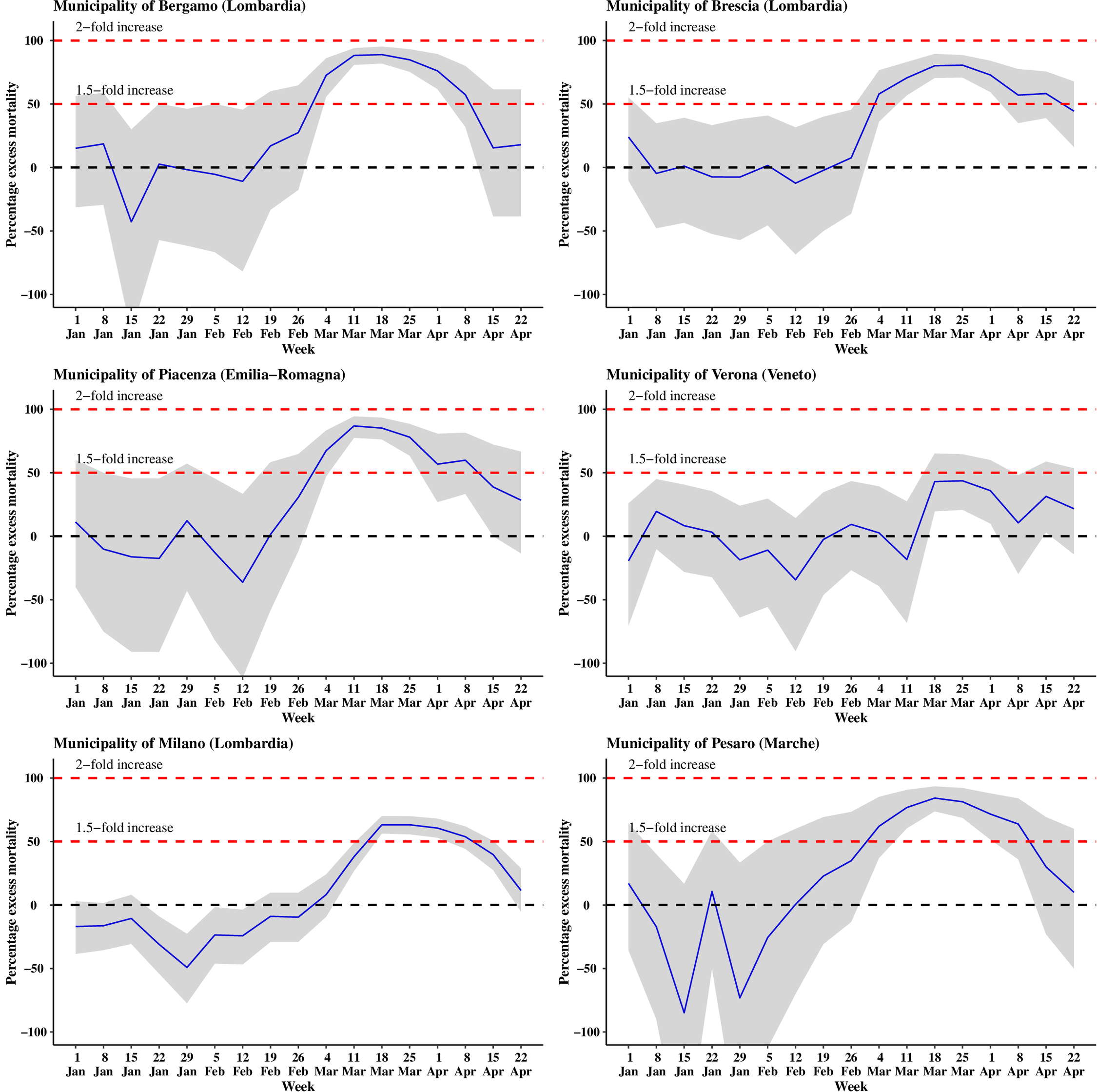

Supplement: S5 Fig — Posterior mean (blue) and 95% interval (gray ribbon). (TIF) [file pone.0240286.s005.tif]

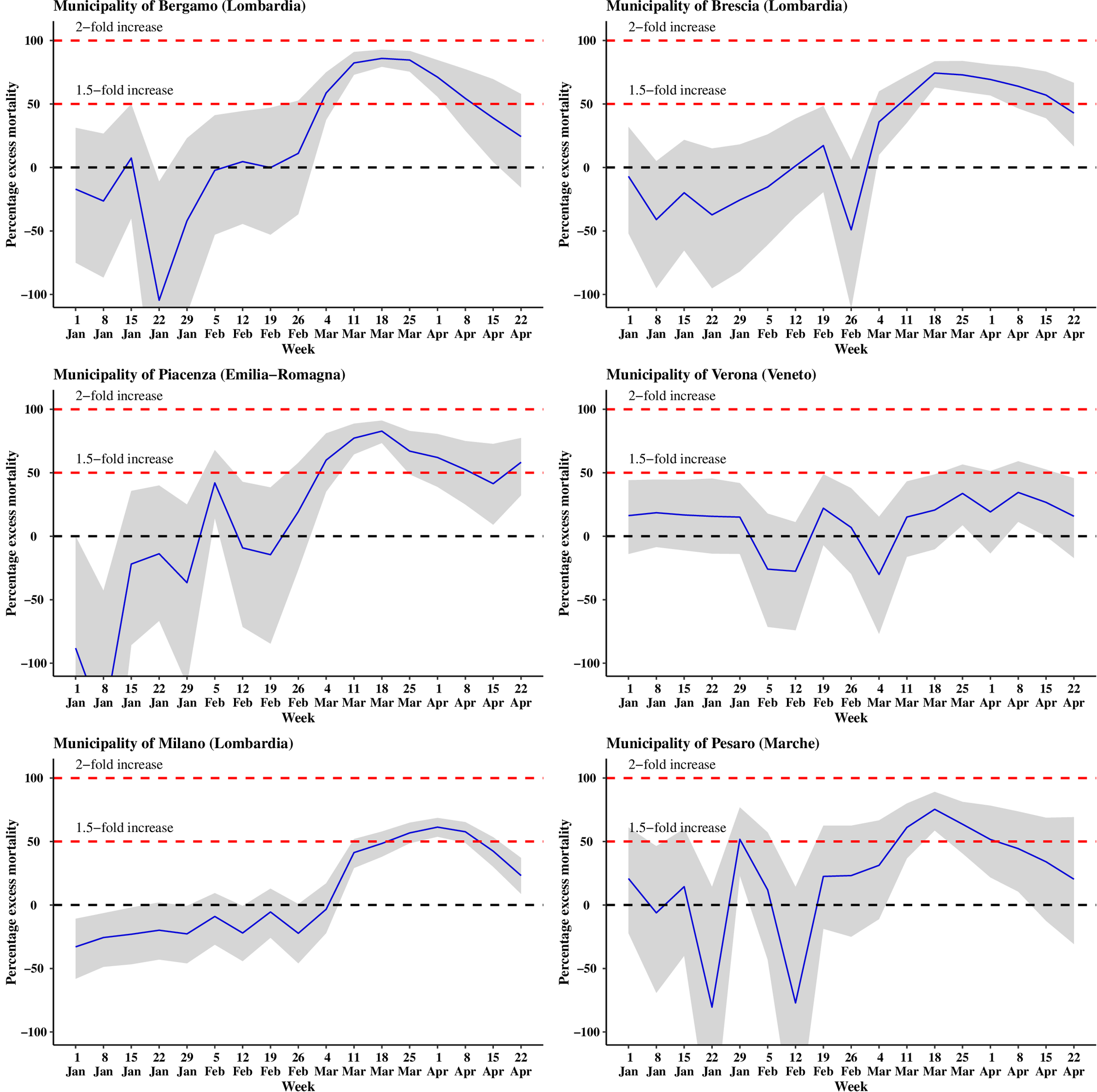

Supplement: S6 Fig — Posterior mean (blue) and 95% interval (gray ribbon). (TIF) [file pone.0240286.s006.tif]
