# Supplementary material for: Estimating weekly excess mortality at sub-national level in Italy during the COVID-19 pandemic
Source: PLoS One. 2020 Oct 9;15(10):e0240286. doi: 10.1371/journal.pone.0240286 (PMC7546500; doi:10.1371/journal.pone.0240286)
Supplement: Attachment — Submitted filename: Responses_to_reviewers.docx [file pone.0240286.s007.docx]

**Responses to reviewer**

We thank the reviewer for the positive comments on the paper. We have provided a point-to-point reply to the requests for clarifications below.

1) This study wasn’t prospectively registered: this aspect should be mentioned among study’s limitations.

**Thanks for this comment. We do not believe that this is a limitation of the study, rather one of its characteristics. In fact, in order to estimate excess mortality, observed deaths are compared with predicted ones. While the predicted one could be estimated prospectively, the comparison can only be done retrospectively. We have added this point to the Discussion, lines 238-240.**

2) The Abstract should clarify that the percent excess in mortality of 84.2% (73.8%-93.4%) for the city of Pesaro refers to males.

**Thanks, we have clarified that the results in the abstract are related to males.**

3) The Authors should provide a reference for the spatiotemporal disease mapping approach at page 2 line 39.

**We moved forward the reference**

**Lawson A, Lee D. Bayesian disease mapping for public health. In: Handbook of**

**statistics. vol. 36. Elsevier; 2017. p. 443-481.**

**which provides a recent overview of methods for disease mapping in a spatial and spatio-temporal framework.**

4) The choice of 2016 as the temporal starting point for time series analyses should be justified.

**Thanks for this comment. The available data were 2015-2019, however, we decided to exclude 2015 as it was an unusual year, being characterised by a much higher number of deaths in the winter due to the lower proportion of people vaccinating against flu during the 2014 autumn. We have added a sentence to explain this on lines 60-62.**

5) Some aspects of the results do not have sufficient comment. The above issue represents the weakest point of the manuscript. More specifically the Authors found that, at municipality level, the cities of Bergamo and Pesaro showed the largest percent excess in mortality. Potential explanations of the observed marked geographical heterogeneity in the pattern of mortality should be provided.

**Thanks for the interesting and challenging comment. At present we believe it is impossible to give definite answer related to why we see such marked geographical variability. It is likely to be a combination of several factors, spanning from environmental, socio-economic and healthcare provision related. Additionally, note that while all-cause mortality data which we use in the paper, are the ideal source of data to answer the question “how many excess deaths are there?”, cause-specific mortality data would be needed to answer the question “why is there a mortality excess?” and such data will not be available until the autumn. We have added a sentence to convene this message in the Discussion, lines 295-302.**
