# Supplementary material for: Estimating weekly excess mortality at sub-national level in Italy during the COVID-19 pandemic
Source: PLoS One. 2020 Oct 9;15(10):e0240286. doi: 10.1371/journal.pone.0240286 (PMC7546500; doi:10.1371/journal.pone.0240286)
Supplement: Attachment — Submitted filename: RepliesReviewers2.docx [file pone.0240286.s008.docx]

Responses to reviewer

We thank the reviewer for the additional comment related to the Conclusions. We have modified it to include main findings and main recommendations.

**Conclusions**

**We have used a Bayesian hierarchical modelling framework to describe the evolution of all-cause mortality in Italy from 2016 to 2020 at municipality level and we have pointed out the geographical and temporal differences in the excess mortality during the COVID-19 pandemic. In particular, we have uncovered a striking geographical pattern across Italy, going from the North (severely affected) to the South (which was almost not at all affected). Moreover, we found that the North-West and North-East regions, especially Lombardia, were characterised by a slow return to the expected mortality rate levels, with a persistent excess of mortality by the end of our observation period. Conversely, the Centre and the South did not show evidence of any difference in comparison to the estimated trends.**

**By highlighting and characterising heterogeneity even between close neighbouring municipalities, our findings suggest the needs of well-targeted responses to COVID-19 pandemic, with flexible and coordinated national and sub-national intervention strategies. They are also particularly valuable for policy makers, when adopting control measures that have the potential of heavily affect the health-care system as well as trigger social and economic consequences.**

**From a methodological point of view, our proposed probabilistic approach represents an effective real-time mortality surveillance tool, which, allowing for a continuous monitoring of localised temporal trends, is able to flag where and when the mortality rates deviate from the expected range, suggesting a successive wave of the pandemic, therefore indicating the necessity of a timely intervention.**
